# The Effects of Sodium Dichloroisocyanurate and Calcium Hydroxide as Intracanal Medicaments on Microhardness and Fracture Resistance of Dentin: An In Vitro Study

**DOI:** 10.1002/cre2.70294

**Published:** 2026-02-03

**Authors:** Fereshte Sobhnamayan, Alireza Adl, Negin Firouzi, Saeed Moravej, Samina Gavahianjahromi

**Affiliations:** ^1^ Department of Endodontics, School of Dentistry Shiraz University of Medical Science Shiraz Fars Iran; ^2^ Department of Endodontics, Biomaterial Research Center, School of Dentistry Shiraz University of Medical Science Shiraz Fars Iran

**Keywords:** calcium hydroxides, dentin, fracture resistance, microhardness, sodium dicholoroisocyanurate

## Abstract

**Objectives:**

The aim of this study was to evaluate the effect of sodium dicholoroisocyanurate (NaDCC) as intracanal medicament on the dentin microhardness and fracture resistance of teeth compared to calcium hydroxide (CH).

**Material and Methods:**

Root canals of mandibular premolars (*n* = 153) were instrumented and randomized into two treatment groups and an untreated control group (*n* = 51). Treatment groups received either NaDCC or CH. After 1 week, 1 month, or 3 months, 17 teeth were randomly selected from each group, and two root cylinders were obtained: one for fracture resistance and the other for microhardness test. Two‐way ANOVA, one‐way ANOVA, and Tukey post hoc tests were used for statistical analysis.

**Results:**

The microhardness and fracture resistance in the control and CH groups were not affected by time (*p* > 0.05). However, NaDCC caused significant decreases in dentin microhardness after 1 month and 3 months (*p* < 0.001) as well as in fracture resistance (*p* < 0.05) after 1 month. The groups comparison at each time point showed no significant differences in microhardness and fracture resistance after 1 week (*p* > 0.05). However, after 1 month, a significant reduction in microhardness and fracture resistance was detected for NaDCC and in the fracture resistance for CH (*p* < 0.05). After 3 months, compared to the control group, lower microhardness in NaDCC and CH groups and lower fracture resistance in CH group were detected (*p* < 0.05).

**Conclusion:**

Short‐term application of NaDCC and CH did not adversely affect dentin microhardness. However, prolonged use reduced dentin microhardness, and both medicaments significantly decreased fracture resistance compared with the control group. Limiting the duration of intracanal medicament application is therefore recommended.

## 1 Introduction

Eliminating bacteria and their byproducts is one of the principal purposes of endodontic treatments, although the anatomical complexities of the root canal system led to challenges in achieving this goal (Wong et al. 2021). Thus, applying antibacterial agents as intracanal medications is being investigated to help accomplish this task (Ordinola‐Zapata et al. 2022). These intracanal medicaments should be efficient against a spectrum of bacterial species (Hegde et al. 2023).

As a traditional intracanal medicament, calcium hydroxide (CH) has a high pH and sufficient bactericidal and bacteriostatic effects. It also has the potential to reduce inflammation by decreasing the levels of pro‐inflammatory cytokines (PICs) and matrix metalloproteinases (MMPs). However, due to the buffering effect of dentin and low diffusion, this medicament has limited effect against bacteria in the depth of dentin (Adl et al. 2014; Adl et al. 2022).

On the other hand, prolonged exposure to CH is related to possible fractures, probably caused by damage to the organic matrix of dentin. CH also degrades the acidic proteins and other dentin proteoglycans necessary to bind the hydroxyapatite crystals to the collagen network (Andreasen et al. 2002; Silva et al. 2023; White et al. 2002). Therefore, the quest for a more suitable intracanal medicament remains a subject of investigation.

Sodium dichloroisocyanurate (NaDCC), a commercially available tablet used in various industries, holds potential as a novel intracanal medicament. Like sodium hypochlorite (NaOCl) the most widely used endodontic irrigant, NaDCC produces hypochlorous acid (HOCl), a well‐known oxidizing agent.

However, NaDCC releases only approximately 50% of its chlorine as free available chlorine (FAC) (Bloomfield and MILES 1979), thus its stabilized chlorine acts as a reservoir of HOCl which is rapidly released when the FAC is depleted (Kuechler 1999). It may also offer other advantages in terms of stability, safety, up‐front cost, and convenience (Clasen and Edmondson 2006).

These unique properties and promising results in other applications pique curiosity about its potential in endodontic treatments (Heling et al. 2001; Rossi‐Fedele et al. 2011).

Heling et al. showed that with equal cytotoxicity, NaDCC was more effective than sodium hypochlorite against Enterococcus faecalis (E. faecalis) and other species associated with root canal infection (Heling et al. 2001). An ex vivo study showed that NaDCC and CH, when used as intracanal medicaments, could eradicate an engineered 3‐species biofilm in single‐rooted teeth in an ex vivo model (Chan et al. 2020). In another study, NaDCC was compared with 2‐hydroxyisocaproic acid and CH plus 2% chlorhexidine as intracanal medicaments in an extracted tooth model against E. faecalis. It showed higher antibacterial efficacy and acted more rapidly than the other two medicaments (Nizar et al. 2025). Moreover, in a recent study, NaDCC presented superior results against E. faecalis and Candida albicans after 3 and 7 days compared to chlorhexidine and CH (Ilango et al. 2025).

Notably, while CH was ineffective against *Candida albicans*, NaDCC demonstrated high efficacy even at lower concentrations (Kurian et al. 2013). This finding underscores the potential of NaDCC as a superior alternative to CH and warrants further investigation as an intracanal medicament. Considering that any intracanal medicament may negatively affect the physical and mechanical properties of radicular dentin, exploring the effect of any new intracanal medicament on the chemical‐physical properties of dentin should be investigated.

No previous study has reported the effect of NaDCC on dentin properties. Thus, this study was designed to evaluate the effect of NaDCC as an intracanal medicament on dentin microhardness and tooth fracture resistance compared to CH.

## 2 Material and Methods

The manuscript of this laboratory study has been written according to Preferred Reporting Items for Laboratory studies in Endodontology (PRILE) 2021 guidelines. Ethical approval was granted for this project by the Ethics Committee of Shiraz University of Medical Sciences (IR.SUMS.DENTAL.REC.1401.044). The samples were accessed for research purposes on June 22, 2022. All data were fully anonymized before accessing and the subject's consent was received for the processing. The data are available in case of sending an official request letter. In the following parts, we will explain the experimental setup preparation and method of analysis step by step in detail. The preparation steps are (A) Sample selections and preparations; (B) Medicament Application; (C) Root sections preparation, and the analysis steps are (A) Hardness test; (B) Fracture resistance test; and (C) Statistical analysis.

### 2.1 Sample Selections and Preparations

In this experimental in‐vitro study, 153 single‐rooted lower premolar teeth were selected. Teeth with cracks, caries, fillings on the root surface, and previous endodontic treatments were excluded from the study. With no previous treatment and no resorptions and calcifications, single canals were confirmed with radiography. Additionally, samples were selected to be almost similar in buccolingual and mesiodistal dimensions (±8%). The storage medium was 0.1% thymol solution at 4°C for 6 months after extraction. The endodontic access cavity was prepared using a round diamond high‐speed bur with air‐water cooling. Working length was determined by observing the tip of a #15 K‐file (Mani Inc. Tachigi‐ken, Japan) just beyond the apical foramen and subtracting 1 mm from the measured length. The canals were prepared using Protaper rotary instruments (Dentsply Maillefer, Ballaigues, Switzerland) up to F4. Canal irrigation was performed between each file using 1.5 mL of NaOCl 5.25% (NikDarman, Tehran, Iran). At the end of preparation, each canal was irrigated with 5 mL NaOCl, 5 mL EDTA (Marvabon, Tehran, Iran), flushed with 5 mL sterile saline, and dried with sterile paper points (DiaDent, Tehran, Iran).

### 2.2 Medicament Application

The samples were randomly allocated into three groups (*n* = 51) based on the type of intra‐canal medicament: NaDCC, CH, and no medicament as the control group. One gram of CH (Merck India Ltd., Mumbai, India) or NaDCC (Merck KGaA, Darmstadt, Germany) powder was mixed with 0.7 mL of saline solution on a sterile slab. Prepared medicaments were applied to the canal spaces with a sterile lentulo spiral (Mani Inc. Tachigi‐ken, Japan) in a slow‐speed handpiece and packed in the canal space to the level of the cemento‐enamel junction using various sizes of sterile pluggers. The remnant medicaments were removed from the pulp chamber using a moist cotton pellet, and the access cavities of all teeth were sealed with a temporary restoration. The samples were stored at 37°C and 100% humidity environment for 1 week, 1 month, or 3 months.

### 2.3 Root Sections Preparation

**Figure 1:**
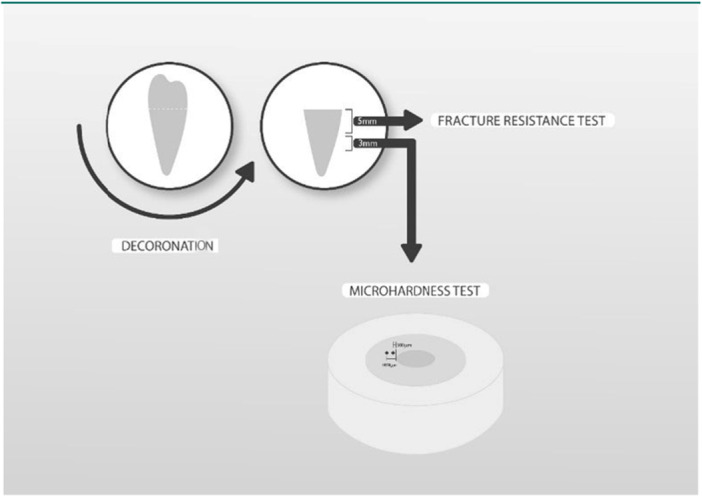
Schematic view of root section preparation.

Root section preparation was adopted from the study by Yassen et al. (Yassen et al. 2013). Seventeen samples were randomly selected in each period. Teeth were decoronated 0.5 below the cemento‐enamel junction using a diamond disk in a low‐speed handpiece and water coolant. Then, each root was horizontally sectioned to obtain two root cylinders: a 5‐mm cervical segment for fracture resistance testing and a 3‐mm mid‐root segment for microhardness measurement (Figure 1). The root cylinders from the treatment groups were irrigated with distilled water to remove the medicaments.

### 2.4 Hardness Test

**Figure 2:**
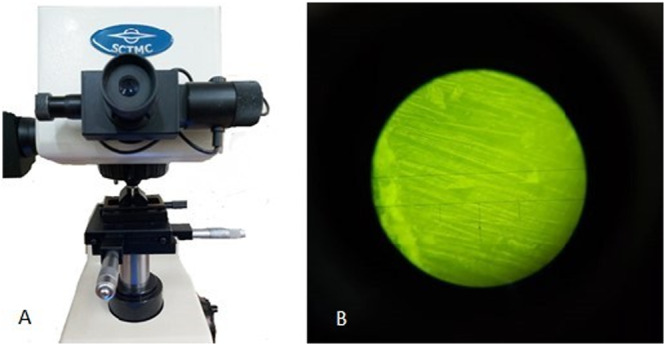
(A) Vickers hardness tester (SCTMC, MHV‐1000Z, China). (B) Microscopic view of Vickers hardness measurement.

The 3‐mm root sections were mounted in acrylic blocks, ensuring their stability, and then the coronal surface was polished with 1000,2000, and 4000‐grit silicon carbide papers. As the final cleansing step, samples were placed in an ultrasonic device and deionized water for 3 min. Polished root sections were then placed in the Vickers Microhardness Tester (SCTMC, MHV‐1000Z, China) (Figure 2). Root surface microhardness was tested at 3 points, 500 micromillimeters from the dentin–pulp interface. A 50‐gr vertical load was applied for 15 s on each point. The notches were observed with a digital optic microscope, and the exact diameter of each notch was measured using image‐analyzed software. The mean results of the three points were concluded as the microhardness of root sections.

### 2.5 Fracture Resistance Test

**Figure 3:**
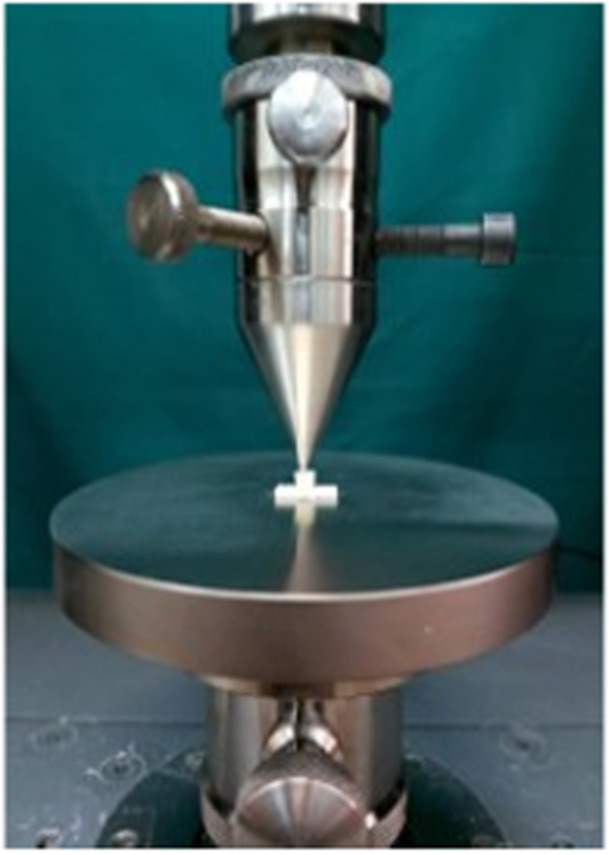
Fracture resistance test universal testing machine (Z020, Zwick, Germany).

Five‐mm root sections were subjected to vertical loading until failure in a universal testing machine (Z020, Zwick, Germany) (Figure 3). First, a seat for the loading arm was made in the coronal part of the root cylinder using a round carbide bur in a low‐speed handpiece. The samples were vertically attached to the lower platform of the device with double‐sided adhesive. A 1.9 mm diameter spherical tip fixture was lowered until it touched the coronal seat, and then vertical forces were applied at a rate of 0.5 mm/s.

A sudden decrease of more than 25% of the load was considered a failure, and the amount of the force was recorded in Newton by the computer connected to the device.

### 2.6 Statistical Analysis

The SPSS software version 22 (IBM Co., Chicago, IL, USA) was used for data analysis. The two‐way ANOVA test measured the interaction of time intervals and groups followed by one‐way AVOVA and Tukey's post hoc test. A statistical significance of 0.05 was applied for the analysis.

## 3 Results

Results for microhardness and fracture resistance tests are mentioned in different parts:

### 3.1 Microhardness

The result of the microhardness test is shown in Table 1.

**Table 1:** Mean (SD) of microhardness for roots treated with endodontic regeneration medicaments and a control group for 1 week, 1 month, and 3 months at 500 micrometer from the pulp–dentin interface.

500 µm from pulp to dentin interface^a^			
Groups	1 Week	1 Month	3 Months
Control	44.93 (3.99) Aa	44.20 (9.88) Aa	47.97 (9.9) Aa
CH	40.52 (6.58) Aa	38.52 (9.27) Aa	37.38 (9.09) Ba
NaDCC	43.68 (8.65) Aa	25.88 (4.33) Bb	36.98 (4.69) Bc

^a^ Different upper‐case letter indicates significant differences between different groups within the same time‐point and different lower‐case letters indicate significant differences between the three time‐points within the same group.

Two‐way ANOVA demonstrated a significant interaction between medicament type and time, indicating that changes in microhardness over time differed among the groups (*p* < 0.001). Therefore, separate one‐way ANOVAs were performed within each time point, followed by Tukey post hoc tests when necessary.

At 1 week, no significant differences were found among the three groups (*p* = 0.151).

At 1 month, the differences were significant (*p* < 0.001), with the NaDCC group exhibiting the lowest microhardness, significantly lower than both the control and CH groups (*p* < 0.001).

At 3 months, significant differences were again observed (*p* < 0.001), with both CH and NaDCC showing significantly reduced microhardness compared with the control group (*p* = 0.002 and *p* = 0.001, respectively).

Comparison of each medicament across different time points showed that time had no significant effect on the control group (*p* = 0.439) or the CH group (*p* = 0.549). However, time had a significant effect on the NaDCC group (*p* < 0.001), with all three time intervals differing significantly from one another (*p* < 0.05). Microhardness was highest at 1 week, followed by 3 months, and lowest at 1 month.

### 3.2 Fracture Resistance

The mean and standard deviation for teeth treated with different medicaments are presented in Table 2. Two‐way ANOVA showed no significant interaction between medicament type and time (*p* = 0.561); therefore, pairwise interaction comparisons were not required.

**Table 2:** Mean (SD) of fracture resistance for roots treated with different medicaments and untreated control group for 1 week, 1 month, and 3 months in Newton.

Groups^a^	1 Weak	1 Month	3 Months
Control	647.64 (253) Aa	459.41 (138.15) Ab	564.29 (121.12) Aa
CH	428.35 (192.32) Ba	369.52 (115.76) Bb	419.52 (64.75) Aa
NaDCC	494.58 (184.78) Ba	379.76 (79.96) Bb	443.88 (160.56) Aa

^a^ Different uper‐case letter indicates significant differences between different groups within the same time point and different lower‐case letters indicates significant differences between the three time‐points within the same group.

Regardless of the time interval, the control group exhibited significantly higher fracture resistance than both the CH and NaDCC groups (*p* < 0.001), while no significant difference was found between the two medicament groups (*p* = 0.278).

Regardless of the medicament used, time had a significant effect on fracture resistance (*p* < 0.001). The 1‐month specimens showed the lowest values, significantly lower than both the 1‐week and 3‐month specimens (*p* = 0.001, *p* = 0.019 respectively), whereas no significant difference was observed between the 1‐week and 3‐month intervals (*p* = 0.125).

## 4 Discussion

NaDCC as an intra canal medicament with appropriate antibacterial efficacy was investigated in the present study to evaluate its effect on the microhardness and fracture resistance of root dentin compared to CH in the present study. Both CH and NaDCC did not significantly affect dentin microhardness after 1 week, which is consistent with the findings of El‐Rashidy et al. (El‐Rashidy et al. 2024). However, our 1‐month results differed from their observations. In our experiment, prolonged exposure to NaDCC led to a significant reduction in microhardness, whereas CH showed a non‐significant effect. A plausible explanation for this discrepancy lies in the methodological differences between the two studies. We applied intracanal medicaments following a clinical protocol inside the root canal system and assessed microhardness at 500 μm from the canal wall after medicament removal. In contrast, El‐Rashidy et al. (El‐Rashidy et al. 2024) used dentin disks directly immersed in the medicaments, exposing a larger surface area to direct chemical interaction, which may have exaggerated the demineralizing potential of CH while underestimating the diffusion‐dependent effect of NaDCC. Therefore, variations in sample preparation, medicament contact area, and simulation of clinical conditions likely contributed to the opposite outcomes observed at the 1‐month evaluation period.

In the current study, NaDCC increased the microhardness of dentin after 3 months, although it did not reach its first microhardness value and in general both CH and NaDCC showed significantly lower microhardness values in 3 months compared to the control group.

An interesting pattern was observed in the NaDCC group, where microhardness values decreased markedly at the 1‐month interval and subsequently increased at 3 months, although they did not return to baseline or match the control group. This non‐linear behavior suggests that the influence of NaDCC on dentin physical properties may not be purely progressive or unidirectional. Instead, the material may induce a dynamic sequence of changes in the dentin substrate, possibly involving an initial phase of demineralization followed by partial remineralization or structural reorganization over time. These findings highlight the possibility of complex and temporally variable interactions between NaDCC and dentin, which warrant further investigation using analytical techniques such as FTIR to elucidate the underlying chemical processes.

In our study, CH demonstrated a reduction in microhardness after 1 month compared with the control group, although this difference did not reach statistical significance. This was in agreement with Pinto et al. investigation that has shown no adverse effect of CH on the microhardness of radicular dentin in 30 days (Pinto et al. 2020). Yassen et al. also showed that using CH in 4 weeks did not reduce the microhardness of dentin compared to TAP and DAP (Yassen et al. 2013). Contrary to the present study, Naseri et al. (Naseri et al. 2019) and Yilmaz et al. (Yilmaz et al. 2016) showed that using CH in 4 weeks significantly decreases the microhardness of root canal dentin. Denaturation of dentin organic matrix due to the alkalinity of CH was explained as a cause of decrease in microhardness value (Naseri et al. 2019).

The controversial results of different studies could be explained by different kinds of CH, different concentrations of this material and even different animal or human root dentin that have been used in these studies.

Regarding fracture resistance, according to the results of two‐way ANOVA, no significant interaction was found between medicament type and time. Therefore, the effects of CH and NaDCC on fracture resistance cannot be interpreted based on the main effects. Independent of time, both CH and NaDCC showed significantly lower fracture resistance compared with the control group, while no difference was observed between the two medicaments. Similarly, the main effect of time was significant, with the 1‐month specimens showing the lowest values, followed by higher values at 1 week and 3 months.

Interestingly, the NaDCC group exhibited a pattern in fracture resistance similar to that observed for microhardness: the lowest values were recorded at 1 month, followed by an increase at 3 months. This similarity suggests that the temporal trend of initial weakening followed by partial recovery may reflect a consistent dynamic effect of NaDCC on dentin properties, even though pairwise comparisons across time points for this medicament are not statistically supported due to the absence of interaction.

The reduction in fracture resistance associated with both medicaments is therefore a general effect, not limited to any specific time point, and the temporary decrease observed at 1 month reflects a time‐related change occurring regardless of the medicament used.

In agreement with the present study, Anderson et al. (Andreasen et al. 2002) showed that applying CH for 2 months showed a significant reduction in the fracture resistance of root dentin. Al‐Hiyasat et al. (Al‐Hiyasat et al. 2021) and yassen et al. (Yassen et al. 2015) also showed an adverse effect of CH on the fracture resistance of dentin in 3 months. On the other hand, Hatibović‐Kofman et al. (Hatibović‐Kofman et al. 2008) and Kahler et al. (Kahler et al. 2018) showed that long‐term use of CH did not show an adverse effect on the fracture resistance of root dentin. The controversial results of these studies could be attributed to the different kinds of teeth that have been selected (animal teeth vs. human teeth, mature teeth vs. immature teeth. On the other hand, different studies used different irrigation protocols or even different kinds of fracture resistance tests. One of the limitations of the present study was that it was conducted on extracted teeth. Under such conditions, factors such as the age of the teeth, the time elapsed since extraction, and the storage conditions may affect the structure of the dentin as well as the interaction between the dentin and the intracanal medicaments.

## 5 Conclusion

Within the limitations of this in vitro study, neither NaDCC nor CH adversely affected dentin microhardness after 1 week of application. However, prolonged use resulted in reduced dentin microhardness, with NaDCC showing earlier effects than CH. Both medicaments significantly reduced fracture resistance compared with the control group, regardless of application time, and the lowest fracture resistance values were observed at the 1‐month interval.

These findings underscore the importance of cautious application durations for intracanal medicaments to minimize the risk of dentin weakening. Further research is recommended to explore the long‐term chemical interactions of NaDCC with dentin and to develop strategies for mitigating its adverse effects during extended usage.

## Author Contributions

Fereshte Sobhnamayan contributed to design the task and idea concept, Alireza Adl contributed as the corresponding author, Negin Firouzi contributed to preparing the experimental setup, Saeed Moravej contributed to run the experimental setup and record the results, Samina Gavahianjahromi contributed to paper preparation and review the paper.

## Ethics Statement

Ethical approval was granted for this project by the Ethics Committee of Shiraz University of Medical Sciences (IR.SUMS.DENTAL. REC.1401.044). The samples were accessed for research purposes on 22/06/2022.

## Conflicts of Interest

The authors declare no conflicts of interest.

## Data Availability

The data that support the findings of this study are available on request from the corresponding author. The data are not publicly available due to privacy or ethical restrictions.
